# 'Innovation policy is a team sport' - insights from non-governmental intermediaries in Canadian innovation ecosystem

**DOI:** 10.1186/s40604-018-0062-8

**Published:** 2018-11-27

**Authors:** Merli Tamtik

**Affiliations:** Department of Educational Administration, Foundations and Psychology, Faculty of Education, University of Manitoba, 263 Education Building, Winnipeg, MB R3T 2N2 Canada

**Keywords:** Innovation ecosystem, Intermediary stakeholders, Non-governmental agents, Canada, 创新生态系统, 中间利益相关者, 非政府代理人, 加拿大, écosystème de l'innovation, acteurs intermédiaires, agents non gouvernementaux, Canada, инновационная экосистема, промежуточные участники, негосударственные участники, Канада, Ecossistema de inovação, stakeholders intermediários, agentes não governamentais, Canadá, ecosistemas de innovación, intermediación, agentes no gubernamentales, Canada

## Abstract

Policy-makers and practitioners alike have increasingly embraced the innovation ecosystem approach to support the flow of knowledge within the Triple Helix framework. This approach focuses on the collaborative and interdependent nature of innovation, which is based on social aspects of knowledge transfer supporting relationships, partnerships, and connections. The important role of intermediary stakeholders that help to facilitate such partnerships is under-researched. This paper examines the work of three intermediary stakeholders in the Canadian innovation ecosystem—the Canadian Science Policy Centre, the MaRS Discovery District, and university Vice Presidents Research. By interviewing 40 experts from the federal and provincial governments, non-governmental organizations, industry, and the higher education sector in Ontario, this study examines how innovation ecosystems are created and what factors influence the success of bringing diverse stakeholders together. The findings suggest that strong political vision and leadership, an inclusive approach to recognizing the needs of diverse stakeholders, and clarity on ways to measure and fund innovation serve as important factors in the Canadian innovation ecosystem.

**Electronic supplementary material:**

The online version of this article (10.1186/s40604-018-0062-8) contains supplementary material, which is available to authorized users.

## 抽象

政策制定者和从业者都越来越多地采用创新生态系统方法来支持三螺旋框架内的知识流动。这种方法侧重于创新的协作和相互依赖的本质,基于支持关系、合伙和联系的知识转移的社会方面。帮助促进此类合伙关系的中间利益者的重要作用尚未得到充分研究。本文考察了加拿大创新生态系统中的三个中间利益相关者的工作 *——*即加拿大科学政策中心、*MaRS*探索区和大学副校长研究。通过采访安大略省的联邦、省政府、非政府组织、工业和高等教育部门的*40*位专家,本研究探讨了创新生态系统怎样被创建,以及什么因素会影响成功地将不同利益相关者聚集在一起。研究结果表明,强有力的政治愿景和领导力,认识到不同利益相关者需要聚在一起的包容态度,以及衡量和资助创新方式的明确性是加拿大创新生态系统的重要因素。

## Résumé

Les décideurs et les praticiens adoptent de plus en plus l'approche par écosystème d'innovation pour soutenir le flux du savoir dans le cadre de la Triple Hélice. Cette approche met l'accent sur la nature collaborative et interdépendante de l'innovation qui repose sur les aspects sociaux du transfert du savoir en soutien aux relations, aux partenariats et aux connexions. Peu d'études sont consacrées au rôle important des acteurs intermédiaires qui facilitent de tels partenariats. L'article examine le travail de trois de ces acteurs intermédiaires dans l'écosystème canadien de l'innovation; il s'agit de Canadian Science Policy Centre, MaRS Discovery District, et University Vice Presidents Research. Quarante experts des gouvernements fédéral et provinciaux, d’organisations non gouvernementales, du monde des affaires et de l'enseignement supérieur dans l'Ontario ont été interrogés. L'étude examine le contexte de création des écosystèmes d’innovation et les facteurs du succès du rapprochement entre divers acteurs. Les résultats suggèrent qu'une vision politique et un leadership forts, une prise en compte des besoins individuels des divers acteurs et la transparence dans la mesure et le financement de l'innovation constituent des facteurs importants dans l'écosystème canadien de l'innovation.

## Abstrato

Legisladores e afins têm, cada vez mais, incluído a abordagem do ecossistema inovação para dar apoio ao fluxo de conhecimento inerente à estrutura de Hélice-Tríplice. Tal abordagem foca na natureza colaborativa e interdependente da inovação, que é baseada nos aspectos sociais da transferência do conhecimento, dando apoio às relações, parcerias e conexões. O papel importante de *stakeholders* intermediários que ajudam a facilitar tais parcerias também é abordado. Este artigo examina o trabalho de três *stakeholders* intermediários no ecossistema canadense de inovação - O “Canadian Science Policy Centre”, o “MaRS Discovery District” e a atuação de Vice-Presidentes de departamentos universitários de pesquisa. Através de entrevistas com 40 especialistas dos governos federal e provinciais canadenses, organizações não governamentais, indústria e o setor de educação superior em Ontário, este estudo examina como ecossistemas de inovação são criados e quais fatores influenciam o êxito em reunir vários *stakeholders*. As descobertas sugerem que visões políticas fortes e liderança, uma abordagem inclusiva para reconhecer as necessidades de diversos stakeholders e clareza nas formas de mensurar e financiar inovação servem como fatores importantes no ecossistema de inovação canadense.

## Аннотация

Чиновники и предприниматели все активнее вовлекаются в инновационную экосистему для обеспечения генерации потока знаний в рамках Тройной спирали. Такой подход основывается на партнерской и взаимозависимой природе инноваций, которые опираются на социальные аспекты трансфера знаний под воздействием сотрудничества, содействия и связей. Значение роли промежуточных участников, способствующих появлению подобных партнерств, мало исследовано. В настоящей статье изучена работа трех промежуточных участников Канадской инновационной экосистемы - Канадский центр научных стратегий, Центр открытий MaRS и Университетские исследования Вице-Президента. В ходе интервью с 40 экспертами, представляющими федеральные и региональные власти, негосударственные организации, промышленность и сектор высшего образования в Онтарио, в контексте данного исследования было изучено то, как создаются инновационные экосистемы и какие факторы оказывают влияние на успешность объединения различных участников воедино. Результаты показывают, что четкая стратегия и лидерство, комплексный подход к потребностям различных участников, а также понимание путей оценки и финансирования инноваций, являются важными факторами в Канадской инновационной системе.

## Resumen

Tanto los responsables de la formulación de políticas públicas como los profesionales han adoptado el enfoque del ecosistema de innovación para administrar el flujo de información en la Triple Hélice. En este enfoque la relación de los actores de la innovación es colaborativa e interdependiente y la transferencia de conocimiento tiene lugar sobre una matriz social de relaciones, asociaciones, y conexiones preexistentes. Sin embargo, estos intermediarios no han sido aún objeto de estudio sistemático. Este artículo examina el trabajo de tres intermediarios en el ecosistema de innovación canadiense: el Canadian Science Policy Center, el MaRS Discovery District y los vicepresidentes de investigación en las universidades. Usando entrevistas a 40 expertos de los gobiernos federal y provinciales, organizaciones no gubernamentales, la industria y el sector de la educación superior en Ontario, este estudio examina cómo se crean los ecosistemas de innovación y qué factores influyen en el éxito de reunir y coordinar a los varios actores del ecosistema de innovación. Nuestros resultados resaltan la importancia de una visión política y un liderazgo sólidos, un enfoque inclusivo para reconocer las necesidades de los diversos participantes, ​​y la claridad en las formas de medir y financiar la innovación.

## Multilingual abstract

Please see Additional file 1 for translation of the abstract into Arabic.

## Introduction

Innovation performance and research commercialization have become the key indicators for assessing and ranking a country’s economic development (OECD 2016). Although placing high in research production, Canada has been struggling with innovation underperformance. Several recent government and academic reports have lamented the modest uptake on research commercialization, the limited emergence of world-class innovation hubs, and the weakness in long-term wealth creation in Canada (Creutzberg 2011; Government of Canada 2016; Schwanen 2017; CCA 2018). The key contributor to Canada’s comparatively low innovation performance tends to be the overall complexity of its innovation system. The diversity of the stakeholders, limited policy coordination between multiple levels of government, and confusion over specific roles and responsibilities have contributed to fragmented approaches in Canadian innovation policy (Niosi 2000; Salazar and Holbrook 2007).

Canadian federalism is part of the problem. Research by Mitchell and Ledwell (2011), Creutzberg (2011), and Sharaput (2012), among others, has emphasized the need for a clearer division of policy roles among the federal and provincial governments in Canada to support innovation. The federal government has an overall responsibility for Canada’s economic competitiveness and social well-being, yet the higher education sector, including its universities, is regulated and governed by the provincial governments. The federal government has an influence over research collaborations through three granting councils—the Social Sciences and Humanities Research Council (SSHRC), the Natural Sciences and Engineering Research Council (NSERC), and the Canadian Institutes of Health Research (CIHR) (Doern 2007). Attempts have been made to foster university-industry connections through specific funding programs (e.g. the SSHCR Partnership Grants). Recently (in 2018) the federal government (Innovation, Science and Economic Development Canada) launched a new “*Innovation Supercluster Initiative”,* investing $950 million in a few selected industry-led consortia across the diverse regions in Canada (Government of Canada web). Canadian provinces have developed their own strategies, programs, and policies that support university-industry linkages. Ontario has an elaborate system of programs that support university-industry knowledge flows operated under the purview of the Ministry of Economic Development and Innovation (MEDI) (Bramwell et al. 2012). Regional innovation initiatives are influenced also by the municipal governments’ support for regional economic development (Currie 2011). These examples suggest a tendency towards fragmented project-based rather than systemic approaches in Canadian innovation policy.

Driven by the idea that innovation often emerges unexpectedly through non-linear networked ways, the developments regarding industry connections in Canada have shifted to local and regional levels. This has given rise to city-regions where a concentration of innovation-focused firms is emerging (Bradford and Bramwell 2014; Wolfe 2014). In particular, Toronto, Montréal, Vancouver, Ottawa, and Calgary have fostered patents and high-tech companies at nearly twice the rate of other cities (CCA 2018). However, these examples tend to point to developments in areas of long-existing economic capacity. The general trend of new entrepreneurial university-industry collaborations is still circumscribed and would benefit from a clear coordinated vision for Canada’s innovation agenda.

In order to support systemic approaches to innovation, Canada needs stronger connections, knowledge flows, and policy coordination among the Triple Helix stakeholder groups—governments, universities, and industry (Fisher, Atkinson-Grosjean and House 2001; Bramwell, Hepburn, and Wolfe 2012). While the government has created several opportunities to bring university and industry stakeholders closer together, there are inherent organizational challenges (different missions, issues around intellectual property ownership) that have created little incentive for partners to collaborate (Bramewell et al., 2012). Thus, there is a need to investigate how Triple Helix connections can be better facilitated and maintained to enhance the opportunities for Canada’s innovation scene. The main argument of this paper suggests that intermediary non-governmental stakeholders can play an increasingly significant role in building connections and potentially enhancing opportunities for more coordinated approaches in the Canadian innovation ecosystem. This paper focuses on the role of The Canadian Science Policy Centre, the MaRS Discovery District, and university Vice Presidents Research in Ontario, Canada, in creating the ecosystem needed for enhanced relationships and innovation capacity. The following research questions guided the study: *What role do individual agents within the Triple Helix framework play in the Canadian innovation landscape? How do they influence university-industry-government-society relationships? What factors support and inhibit their work?*

## Conceptual framework

The concept of the Triple Helix (Etzkowitz and Leydesdorff 2000) provided the foundation for understanding the interactions between government, businesses, and industry in this study. The Triple Helix’s core thesis suggests that the potential for innovation lies in the hybridization of elements from university, industry, and government to generate new institutional and social forms for knowledge (Ranga and Etzkowitch 2015). The Triple Helix concept is a useful lens as it accommodates both institutional and individual roles within the knowledge triangle, recognizing the importance of organizational (MaRS, Canadian Science Policy Centre) and individual actors (Vice Presidents Research) in creating new types of linkages within the Helix. This approach fits with an understanding of the nature of innovation as a non-linear, networked, interactive process, often involving an unpredictable combination of research and action by key individuals, universities, businesses, and governments (Doern, Phillips, and Castle 2016).

Recent research on innovation has begun to emphasize the *innovation ecosystem approach* (Jackson 2011; Mercan and Goktas 2011; Bramwell et al. 2012). This approach focuses on the interdependent and constantly evolving relationships between a wide spectrum of partners and draws attention to how their interactions affect knowledge creation and uptake. Innovation ecosystems embody an organic and holistic bottom-up approach to economic development that supports innovation. Innovation ecosystems consist of individuals, communities, organizations, material resources, rules, and policies across large and small businesses, universities, colleges, government, research institutes and labs, and financial markets that collectively work towards enabling knowledge flows (Bramwell et al. 2012). This approach emphasizes the contributions of individual actors and institutional structures to enhance institutional innovation capacity within the broader Triple Helix framework (Gertler 2010). These interactions can emerge spontaneously; however, scholars have pointed to the importance of focused social roles and the intermediary activities that play a part in the process (Morgan 2002; Kitagawa 2004). Researchers have argued that maintaining relationships through processes of learning and communication, creating networks of institutions, and building up ‘social capital’ by linking local partners to resources might be the keys to innovation success (Kitagawa 2004; Wolfe and Nelles 2008). Kitagawa (2004) pointed to the presence of institutional ‘nodal points’ (individuals or organizations) and the physical and communicative proximity of actors as key factors for innovation network development. More recent scholarship has focused on the role of community actors and local civic groups as intermediaries in working with municipal and regional governments to support innovation (Wolfe, 2010; Wolfe and Bramwell 2016; Bramwell and Pierre 2017).

These individual agents are often conceptualized as ‘boundary-spanners’, ‘knowledge brokers’, or ‘intermediary actors’ (Howells 2006; Bielak et al. 2008; Meyer 2010), to identify a range of stakeholders involved in mediating activities in the innovation process. Cooper (2012) identified three knowledge mobilization strategies that intermediaries use to help communicate and distribute information across stakeholder groups—utilizing products, creating events, and strengthening networks. The use and creation of *products* includes disseminating research reports (long and short versions), adapting products tailored for specific audiences (tool kits for practitioners, briefs for policymakers), and creating systematic reviews, policy-related documents, terms/glossaries, reference lists, and annotated bibliographies. Organizing *events* helps to bring otherwise distant stakeholders together for sharing information and forming new connections. Conferences, professional development workshops, and training videos are often used. *Networks* include facilitating dialog and user engagement among stakeholders with the help of technology (blogs, Facebook, list serves, wikis) and circulating resources and disseminating research. Howells (2006) proposed a total of ten strategies intermediaries utilize, including identifying potential partners, foresight and diagnostic work, helping to combine the knowledge of partners, facilitating contact, independent advice and mentoring on protecting intellectual property, and evaluation of the outcomes of innovation collaboration. The Triple Helix literature has been largely silent on and is in need of a deeper understanding of the role of intermediaries (Todeva 2013, Bellgardt et al. 2014, Havas 2015). By addressing this gap in the literature within the Canadian context, this paper seeks to contribute to the broader Triple Helix literature on the work of non-governmental intermediaries.

## Research methods

In order to answer the research questions, a two-phase case study approach was adopted, focusing on the province of Ontario and including the Canadian federal government. Ontario was chosen as the top-performing province in research and innovation in Canada (Conference Board of Canada 2015). The objective of the first phase was to conduct an environmental scan and identify relevant individuals, organizations, initiatives, and policy documents associated with Canadian innovation policy. A content analysis (Weber 1996) of 15 policy documents, including the most recent federal and provincial government strategies, policy reports, and analyses, was carried out. The goal was to examine how policy coordination is understood, who are the key agents in the Canadian innovation system, what strategic initiatives have been taken, and what mechanisms have been applied to support innovation.

The objective of the second phase was to collect personal experiences from the stakeholders involved in the Canadian innovation system to examine their role, their potential impact on innovation policy, and their opinions on factors helping or hindering their work. The data were collected as part of a larger SSHRC-funded study titled “Policy Coordination in Canadian Innovation Policy”. Interviewees were selected based on their association with the main Triple Helix groups—government, industry, and university—with snowball sampling (Biernacki and Waldorf 1981) applied to include additional interviewees with relevant expertise. The final sampling frame involved 40 participants representing the following stakeholders: five federal-level government officials (Industry Canada (four), Federal Economic Development Agency for Southern Ontario (one)); 10 provincial-level government officials (Ministry of Economic Development, Employment and Infrastructure/MRI (six); Ministry of Training, Colleges and Universities (three); Cabinet Office/Intergovernmental Affairs (one)); five representatives from the research granting councils (SSHRC (one), NSERC (one), CIHR (one), CFI (one), NRC (one)); five industry representatives (IBM (three), Parteq Innovations (one), Cisco Systems Canada (one)); nine Vice Presidents Research and one senior administrator from the university/college sector in Ontario (Carleton University, University of Guelph, McMaster University, Ryerson University, Seneca College, University of Ontario Institute of Technology, University of Waterloo, Western University, University of Ottawa, and York University) and five non-governmental organization leaders (Genome Canada (one), Ontario Centres of Excellence (one), MaRS Discovery District (one), Canadian Science Policy Centre (one), Canadian Science Advisor at British Commission (one)). For this paper, 11 interviews from the primary sources (Canadian Science Policy Centre, MaRS, and Vice Presidents Research) were included. The experiences of other stakeholders (insights from the federal and provincial governments, research agency representatives, and industry stakeholders) were included to verify the points raised in the primary interviews. All interviews were recorded and transcribed. Data were coded using NVivo software. The analysis involved determining categorical themes (open coding), establishing patterns (axial coding and selective coding), and developing generalizations from the information provided through the interviews (Creswell 1998).

## Results

The findings were categorized and presented based on the main research questions examining 1) the role, 2) the impact, and 3) factors that support or inhibit the work of non-governmental intermediary agents in the innovation ecosystem. The following non-governmental intermediaries were selected as the cases - The Canadian Science Policy Centre, the MaRS Discovery District, and the university Vice Presidents Research of Ontario higher education institutions. The selection was made based on the following criteria – 1) their growing presence in the Canadian innovation scene as attested by the other stakeholders (federal and provincial governments, industry) in the interviews and policy documents; 2) the inclusion of intermediary activities in their mission (e.g. connecting partners, building capacity for policy and practice); 3) a location in Ontario, yet an active connection to all Triple Helix groups. This selection helped to analyze only the data directly relevant to the intermediary activities attested by the interviewees.

### Non-governmental agents of change

#### Canadian Science Policy Centre

Canadian science policy, as well as innovation policy, has been characterized by limited coordination across stakeholder groups (Tamtik 2016; Goracinova et al. 2017). A unique non-government stakeholder that has made a significant mark in promoting science, connecting scientists and policy makers, and advocating for a greater presence of science policy among the federal and provincial governments is the Canadian Science Policy Centre (CSPC). It is a grass-root formed Toronto-based independent organization, founded by an individual Mehrdad Hariri in 2009. Between 2013 and 2018 CSPC was hosted by Ryerson University. It governance structure includes Board of Directors (of six members), led by the founder who also serves as a President and Chief Executive Officer with the help of many volunteers from across the country. The Centre does not have an annual budget allocated to its activities as it operates based on sponsorship money and registration income generated through organizing an annual conference. The Centre’s mission is to create an “inclusive hub for connectivity, convening, capacity-building and catalyzing research in support of effective science policy community” (CSPC web). An interview participant from the Centre explained their primary purpose of functioning as an intermediary in science policy:

“*We try to establish a national dialogue on science, technology and innovation policy across sectors and disciplines because, as you may know, the different sectors sometimes have difficulties to communicate when they differ in their mindset, their language, their technology, etc., their connectivity...We try to strengthen the linkages across various stakeholders of science enterprise.”*

Playing an intermediary role, the Centre has built a significant social capital in mediating among all three Triple Helix pillars—university, industry, and government. Over the years, the Centre has facilitated communication through its newsletters, lecture series, panel sessions, webinars, and its Youtube channel featuring science and innovation policy developments relevant to industry-government-university and highlights of research innovations (CSPC 2018). The annual Science Policy Conference is the most prominent evidence of its intermediary activities, as panels of the conference are organized by presenters from each of the Triple Helix groups—academia, industry, and government. Since 2015 the conference has been strategically held in Ottawa, to secure the attendance of busy government officials who work on Parliament Hill in close proximity, also by being held in the capital, it makes it more accessible and neutral for the participants from other parts of the country to attend. By gradually growing its size, the Centre has managed to grow its visibility and reputation among the politicians that often make an appearance at the conference. Another key function and a guiding principal of the Centre is youth engagement. To further this goal, the Centre has initiated the first and only Science Policy Awards of Excellence in Canada for youth. The Centre is planning to start a Mentorship program where students, early career professionals, and new immigrants who are interested in science and innovation policy will be linked with Canadian science policy experts to facilitate connections and enhance careers. With all these activities, the Centre serves as a flagship in building and maintaining collaborative stakeholder relationships in the Canadian science and innovation landscape.

#### MaRS Discovery District

Another significant player that supports primarily university-industry connections but, government ones as well to a lesser extent is the MaRS Discovery District. It is Canada’s largest non-profit innovation hub, located in Toronto, and home to more than 150 organizations drawing over 6000 people to work there every day (MaRS 2017a). MaRS’s intermediary role is articulated in its mission of being “*an entrepreneurial venture designed to bridge the gap between what people need and what governments can provide*” (MaRS web). This innovation hub was initiated and funded primarily by the Ontario provincial government^1^ in 2005 with the federal government providing additional revenues for its infrastructure. As such, MaRS is an example of government being the creator of a boundary-spanning mechanism that facilitates academic-industry relations (Todeva 2013). MaRS stands for ‘Medical and Related Sciences’, which summarized its early organizational focus. Currently MaRS’s scope has widened to four industries: social innovation; life sciences and health care; information technology, communications, and entertainment (ICE); and physical science, engineering, and “cleantech” (Slaughter 2014). As a registered charity, funding for MaRS comes from corporate sponsors, private donors, support from the provincial and federal governments, and revenue from events held inside the facility.

Different from CSPS, MaRS’s primary capital is economic in nature as it provides (venture) capital helping individuals to launch and grow companies through grants, training programs, and rental space in their downtown Toronto building. Similar to CSPC, its strengths lie also in social capital, as its mission is to build connections between people and physically bring together entrepreneurs, educators, researchers, and business experts. It is governed by a Board of Directors, with the President of the University of Toronto and a professor from Western University included among the corporate board members. With reported revenues of $50 million in 2017, it brings together a mix of tenants including new starts-ups, already established global tech leaders like Facebook, Autodesk, Airbnb, and PayPal, and scientists from the higher education sector (MaRS 2017b).

While MaRS representatives work closely with private sponsors, industry, and both the federal and the provincial governments, the interviewee from MaRS emphasized its essential connections with the municipal government of Toronto. As MaRS is the urban innovation hub in the city of Toronto, it is crucial that the political leadership supports and promotes its work. The participant noted:

“*We work very closely with the municipal government, not as a funder per se, but as a collaborator within the innovation ecosystem in Toronto*...*It may be municipal policies, which give favourable zoning support or lower commercial tax rates to innovation centers.”*

By building a connection with the university sector and making start-up activities tangible and desirable among individuals, MaRS plays an important role in Ontario’s innovation system (Currie 2011). Through collaborative relationships, MaRS provides a formal platform for linking individual entrepreneurs with other Triple Helix stakeholder groups.

#### University vice presidents research

Increasing research evidence demonstrates that individuals within the university sector, such as rectors, presidents, and vice-rectors or vice-presidents, are becoming important players in innovation policy, actively advocating for new industry partnerships, creating regional innovation networks, and attracting talent to local economies (see Benneworth and Charles 2005; Bramwell and Wolfe 2008; Lehmann 2015). Canadian Vice Presidents (VPs) Research are no different as they play a significant advocacy role in the Canadian innovation system (Tamtik 2018).

Within the organizational structure of the Canadian post-secondary education sector, Vice Presidents Research (VPs Research) are top-level administrators whose primary role is to develop and strengthen institutional research capacity. Of the three cases, the work of university VPs Research tends to focus most closely on political relationships with the federal and provincial governments. While their work involves close communication with the industry sector, the informants tended to reflect on their advocacy activities with governments. Vice Presidents described their responsibilities often in regards to strategic participation in external policy processes (“*I have to act as an advocate for research outside the university*”, “*We try to influence the policy objectives*”, “*One of my roles is to be an advocate for Canadian innovation and research policy*”).

The representatives from the federal government as well as granting councils confirmed that university leaders have become significant stakeholders in shaping the innovation agenda. The granting council administrators noted that their policy directions are increasingly influenced by bottom-up initiatives coming from the research institutions. Such broad recognition confirms the advocacy and intermediary role university VPs Research play in Canada’s innovation agenda.

### Evidence of influence

There are several ways to demonstrate evidence of direct outcomes of the intermediary activities examined in this study. For the Canadian Science Policy Centre, the significant evidence of their success in increasing networks has been the growing number of participants in their annual conference and gradually increasing number of subscribers to their social media channels. For example, at its most recent Science Policy Conference there were 677 registered participants representing mainly the decision makers and influencers of policy with their organizations, as 23% of them are executives (CEO, Presidents VPs, Executive Directors) and 24% from senior management (CSPC 2018). The dramatic increase in the number of graduate students and post-doctoral students attending the Science Policy Conference in 2017, from 7% to 22% (CCSP 2018), is one indicator that demonstrates the impact of its community-building work. A youth participant reflected: “*There is currently no or very limited, let’s say, channels for the younger generation scientists, graduate students to be introduced to science policies*” (CSPC 2018, p. 6). The conference participants have included high-level government officials and politicians including the Minister of Science, Canada’s Chief Science Advisor, and the Governor General of Canada. In addition, there has been external sponsorship money attributed to their activities. In 2018, the *Fonds de recherche du Québec’s* (FRQ 2018), a provincial research funding agency, announced their support for the Centre over five years to further enhance Québec’s participation in advancing Canadian science policy. Another attestation of the Centre’s influence is the recent project in partnership with the Chief Science Advisor Dr. Mona Nemer (@ChiefSciCan, Tweet) titled “Science Meets Parliament” that brings scientists working in Canada to Parliament Hill in order to attend committee meetings, discuss scientific research, and gain familiarity with the political process. All those activities are crucial for popularizing science and building a coherent community around the topics relevant to science and innovation policy.

MaRS is regularly collecting and reporting on the impact of its activities, measuring capital raised, revenue generated, and the number of people involved in MaRS-supported activities (MaRS 2017b). One of its key roles is maintaining close linkages with industry and other private partners to secure private investments for its capital programs. The pool of private donors investing funds through MaRS include CIBC, Microsoft, Thomson Reuters, The Rockefeller Foundation, and the RBC Foundation (Slaughter 2014). As noted by Currie (2011), MaRS’ activities have put Canada on the international map. While mostly focused on university-business connections, MaRS has had a role in policy development as well. The representative from the MaRS innovation hub noted MaRS’ role in providing feedback to the government:

*“When the government is doing a review of existing policies or existing programs in innovation, we’re often consulted./.../ MaRS is a very large player, I suppose, within Ontario’s ecosystem. So we’re consulted on an ongoing basis about the operations of our programs, about the emergence of new programs and the desirability of those.”*

Three provincial government officials confirmed the role of MaRS in connecting industry and private partners in government-initiated projects. One reflected on the Advanced Computing project: “*Through MaRS and through other channels we’re trying to get small, medium and large businesses to uptake big data practices and make use of advanced computing.*” Another government administrator noted: “*They [MaRS] bring together investors and entrepreneurs, mentors, business advisors, people with business ideas./.../The aim for companies is to be innovative for investment and innovation.*”

MaRS experts have been involved in provincial program evaluations, which have been used as guidelines for the next steps in provincial innovation policy: “*In 2012 and 2013 we undertook a major strategic review of the policies and programs in innovation within the Toronto region.*” Another example involved establishing decisions for a new entrepreneurship center in Ontario to support innovation, partnering with a Ministry, and providing expert advice on which centers should be established across the province.

The university Vice Presidents Research commented on successful advocacy activities that have led to significant funding increases through establishing new grant programs for innovation to secure economic advantage for Canada. One specific example was the creation of the Canada First Research Excellence Fund (CFREF). The proposals need to demonstrate partnerships with the private sector, international research institutions, and academic organizations with a focus on commercial endeavors and mobilizing research discoveries. The CFREF is a $1.5 billion dollar investment, announced by the federal government in 2014, that addresses the need for Canada’s research-intensive universities to compete on the world stage and attract research talent (Canada First Research Excellence Fund [CFREF] 2017). According to the participants, the fund was created in response to collective lobbying from university leaders representing the top 15 Canadian research universities (U15). Initiating such a program also aligned with the federal elections in 2015, where the federal government was eager to establish a flagship program supporting innovation in Canada. A college sector representative described a different collective advocacy success story, initiated through Polytechnics Canada, which has led to the creation of a specific ‘college only’ research funding category within the NSERC funding scheme. According to the informant, it took them about eight years from the early negotiations to the final outcome of creating this ‘Community and Colleges Innovation Program’. In those cases, the informants noted that factors that they believed led to success included collaborating together as research universities and delivering a consistent and unified message to the federal government. The role of industry in facilitating the Triple Helix relationships of the Vice Presidents tends to be significant. On the one hand, establishing new and maintaining old relationships with industry is crucial for institutional innovation agenda. However, sometimes a tokenistic approach is applied in regards to industry when lobbying the government. An interviewee reflected:

*“All we do is bring industry partners who we have served and we put them in front of a bunch of politicians, both provincial and federal, and they tell their stories. That’s a way to get industry involved, but absolutely there is no way that you could say that the private sector is putting a lot of time and energy into influencing or improving innovation research policy in Canada. I just don’t think that’s true.”*

An industry representative from IBM noted that they get involved in academic partnerships only because government is supportive and expects such an involvement. Those statements indicate the importance of political support in university-industry relationships. Intermediaries then can use it as leverage in their work to facilitate relationships among the Triple Helix stakeholders.

### Factors that support and inhibit intermediary activities

The participants highlighted several factors that tended to have an influence on their work as intermediaries. Political priority allocated to research and innovation activities was the theme most often mentioned. Operational processes are greatly enhanced by having politicians and other high-power government officials involved. Such participation serves as a signal to others that the issues are important and worthy of attention. For example, a government official noted that “*If you get a minister involved, you usually don’t have a lot of problem getting people’s cooperation*”. However, in Canada, innovation and research policy in general has not been a highly visible topic in policy debates. Innovation policy is not a topic that is typically used in political campaigns to attract voters’ attention. Another interviewee from the federal government characterized the process as follows: “*Political stars have to align in order to get everyone working with the same interests and energy levels*.” Several informants mentioned the absence of a clear mandate for innovation policy as an inhibiting factor in Canada. A university Vice President noted: “We need one captain; we don’t need four of them.”

The intermediary organizations described how they would take action when high-level political representation was close by. One informant talked about his proactive strategies:

“*For example, big city mayors meet in Toronto to talk about infrastructure and transportation. What I would do is add innovation to the agenda because that way you can create a common framework for thinking about these kinds of issues. If a federal government won’t do it or can’t do it, then there are other ways in which it can be done. What it involves is having a big enough constituency of players who can make a difference to actually get together to want to do some things.”*

Political priority is important as it is associated with the financial supports essential for creating a well-functioning innovation system.

A representative from the Canadian Science Policy Centre emphasized the importance of communication and the inclusion of a variety of partners for building trust and strengthening working relations among industry, university, and government partners. The participant talked about situations where a government has moved forward with initiatives without a proper consultation process and caused stakeholders’ resistance as a response. He noted:

*“So they [the federal government] did something on their own without proper consultation and inclusion of other stakeholders and the results may not have been necessarily positive. Inclusion of the stakeholders is always important, so that everybody is informed, everybody is included in the process, included, being considered, and heard.”*

Similarly, a participant from MaRS highlighted communication and the inclusion of partners in the process:

“*Innovation policy is a team sport and in order to get better innovation outcomes, you need to link the various players in the ecosystem effectively. That means you have to look at conductivity, you have to look at communication, you have to look at partnership collaboration and you have to ceaselessly promote it.”*

The key obstacle that most informants pointed out was a systemic issue: diverse stakeholders with diverse interests across policy sectors and government levels complicate the creation of one coherent ecosystem of innovation. A MaRS informant summarized the challenge:

*“Factors that inhibit the system are size and diversity and frankly the fact that it’s pretty confusing. I mean, Canada isn’t all that big of a country but we have way too many, it seems, too many places where policy is developed and managed and the proliferation of actors within the system, all with similar kind of objections, creates, I think, unnecessary competition. Innovation seems to be the responsibility of many and therefore the stewardship of no one when it comes to policy. I think that’s a big problem.”*

The participants agreed that the complexity of the innovation system within the governments has often served as a barrier to utilizing a more coordinated approach to activities. According to most of the interviewees, the key challenge in policy support was a lack of vertical communication—collaboration between provincial and federal governments. Provincial policy officers emphasized that stronger coordination with the federal government was needed in terms of data sharing, making decisions on larger capital investments, and collaborating on decisions over broader national priorities.

A strategy that has been helpful among the VPs Research is to create alliances among stakeholders that share common interests. A participant described policy coordination as “*a means of coordinating the development of policy in a manner that is nationally beneficial to the broadest number of stakeholders*”. The most commonly mentioned approach for getting a unified message to the government was to use professional (university/college) associations and other organizations that are increasingly involved in innovation policy debates. Organizations such as the U15 (Group of Canadian Research Universities), the Association of Universities and Colleges Canada (AUCC) (now Universities Canada), the Ontario Council on University Research (OCUR), Colleges Ontario, and Polytechnics Canada were mentioned most often. In order to advocate for innovation policy, there needs to be a clear national-level vision, and common understanding to make effective connections among stakeholders.

Another condition that emerged from the interviews that inhibits long-term intermediary activities was clarity in measuring innovation and its impact. The traditional performance indicators such as R&R expenditure, patents and scientific article count have its flaws (Schwanen 2017), while innovation in social spheres with high impact on societal groups especially in terms of job creation might not be counted. Unless there is clarity on what the expected end goal is and how to measure it, the work of intermediaries has limited focus. An informant pointed to the current weaknesses in the Canadian approach:

*“Our policy framework around measuring impact is weak and it creates a problem for all of us. It probably means there’s money invested in areas where it shouldn’t be because no one is really measuring or they don’t have an ability to measure and areas of synergy aren’t identified because people can’t see the system overall to know which parts of the system when connected work really, really well and what parts don’t.”*

Two provincial government representatives recognized that there is currently no clear way of measuring policy coordination within the innovation system. One of the main indicators for assessing innovation remains expenditure on R&D through funding that has been distributed through research councils or provincial programs. The question of whether the money has been allocated towards supporting an emerging ecosystem or limited project-based initiatives remains unclear.

Not all coordination initiatives related to research funding have led to success stories. There were several examples mentioned, mainly to do with cross-provincial infrastructure projects for research, that are still works in progress. One example was brought to illustrate the limiting eligibility criteria regarding industry partners established by the government in order to apply for funding. Universities are required to partner only with Canada-based industry branches, excluding international industry partners. University leaders frequently shared how limited cross-provincial policy coordination has forced them to reconsider major innovative research collaborations and become more active in advocating for collective interests:

*“There’s an opportunity to build cyber infrastructure in the North. We’re working with a bunch of industry partners across the country. We wanted to implement a program that involves Yukon, Nunavut, Northern Ontario, Northern Labrador. The province of Ontario only wants to support any implementation in Ontario, while it might be a provincial priority in Ontario and therefore Quebec doesn’t want it. So what do you do, skip over that province because it’s not a provincial priority there?”*

The findings demonstrate that there are several operational factors that influence intermediary activities. Political priority, inclusion of partners and clear policy coordination mechanisms would help to enhance the connections within the Triple Helix system. Exclusion of partners, ignoring the diverse interests of stakeholders and lack of clarity for a unified vision for innovation agenda serve as some of the inhibiting factors in intermediary activities. Institutional memory and knowledge on how to handle such increasingly complex system with diverse interest becomes essential.

## Conclusion and discussion

This paper contributes to this limited literature of intermediaries (Bellgardt et al. 2014) in the Triple Helix framework from the Canadian perspective by focusing on the strategies and mechanisms used by three different types of non-governmental intermediaries—an independent non-governmental organization (Canadian Science Policy Centre); Canada’s largest government-initiated innovation hub (MaRS), and influential individuals within research universities (Vice Presidents Research). Following Todeva’s (2013) categorization of coordination activities, the intermediary activities of the three Canadian players represent three distinct approaches to coordination activities. CSPC can be regarded as an example of *network coordination* where platform-based strategies (an annual conference, social media platforms and a comprehensive website) are used for sharing resources and benefits to facilitate communication among the Triple Helix partners. MaRS’s activities can be regarded as an example of *cooperative coordination* whereby partnerships between autonomous organizations (businesses, industry partners, and universities) are facilitated through the sharing of resources and benefits (venture capital, workshops and training programs). University Vice Presidents Research make up the group that primarily uses *political coordination*, whereby they utilize collective membership approaches and alliance types of activities with other universities and industry partners to allocate resources according to their political objectives.

While each of these stakeholders navigates within the Triple Helix framework, the nature of their roles and strategies used are quite different. Each has a unique impact on the overall innovation ecosystem in Canada. The Canadian Science Policy Centre plays the most formal role in bringing the university-industry-government partners together through their annual conference. While not directly affecting the commercialization aspect of innovation, it plays a crucial role in providing opportunities for forming new networks, lobbying the government for supportive science policies, and creating community around innovation policy. The growing presence of politicians and high-level government officials at the Science Policy Conference has increased the visibility of this policy sector among the general public through increased Twitter followers, media coverage, and increased sponsorship. Most importantly, the Centre plays a crucial role in popularizing science and innovation policy among youth, which can have a long-term impact on Canadian innovation policy in the future. The centre has trained more than 700 volunteers since its inception, which has been the only vehicle at this scale for younger generation of scientists and policy makers to be trained in science policy. The MaRS Discovery District serves a direct role in supporting innovation uptake. Through funding opportunities, workshops, and infrastructure, it links emerging entrepreneurs with the knowledge and investors needed for emerging innovations. At the same time, through its strategic relationships with the municipal and provincial governments, it has become a source for relevant feedback and advice for policy development. The role of individual VPs Research is most importantly that of policy advocate to the federal and the provincial governments. While there is active two-way communication between the two governments (federal and provincial) and the research institutions, there is limited collaboration between the provincial and federal governments. University VPs often function as mediators between them, aiming to reconcile diverse interests for increased policy coordination. All three stakeholders mentioned limited political priority and a lack of a clear national vision as a common obstacle in their work.

As Bramwell et al. (2012) noted, innovation ecosystems need networks, capital, representation, knowledge, services, and public support to function effectively. The three stakeholders described in this paper are examples of intermediary actors that provide those components for the Canadian innovation ecosystem. One commonality among these stakeholders is the physical proximity that they all need to succeed in their work. Bringing partners physically together—through a conference, though a rental space, or through a professional organization—is a necessary condition for building relationships. Another core factor that supports their work is a strong political leadership for innovation policy. In Canada, this has been an issue. The decentralized nature of innovation policy creates competition of visions whereby the provincial governments’ agendas are driven by their regional strengths, and the federal government oversees the larger economic growth of the country (see Clowater 2012). This aligns with Ranga and Etzkowitch (2013) who have pointed to the task barriers and relationship barriers within the Triple Helix framework. In the Canadian case, it is the relationship barriers that dominate and are often influenced by imbalances in the power positions within Triple Helix stakeholders (van Geenhuizen 2016). Possession of political power has the ultimate impact on the innovation ecosystem as it influences funding, strategic priorities, and the legitimacy of the intermediaries. Fuerlinger et al. (2015) pointed to the need for governments to create clarity in regulatory environments (e.g. immigration laws, labour rights, business formation process), support entrepreneurship education, especially tertiary education, and publicly fund programs that support the early stages of new ventures. The core inhibitor seems to be the diverse pool of innovation stakeholders that lacks a clear vision for the country. The participants pointed to an inclusive approach to recognizing the diverse needs of stakeholders as a condition of successful intermediary activity. As van Geenuizen, Ye, and Taheri (2016) noted, partner diversity seems necessary to connect and bring together a meaningful set of partners in a mediating role. If there is enough transparency in decision-making and the recognition of diverse needs, it helps to build trust and ease the flow of knowledge in the system. While there are still improvements to be made in Canada on how to prioritize and fund innovation, the three non-government stakeholders examined in this study help to inform our understanding of how to build stronger connections among the Triple Helix stakeholders, creating a dynamic community of innovation.

## Additional files

Additional file 1: Translation of the abstract into Arabic.(PDF 41 kb)

## Abbreviations

AUCC: Association of Universities and Colleges Canada, now Universities Canada
CFI: Canada Foundation for Innovation
CFREF: Canada First Research Excellence Fund
CIHR: Canadian Institutes of Health Research
CSPC: Canadian Science Policy Centre
IBM: International Business Machines Corporation
MaRS: Stems originally from Medical and Related Sciences
MRI: Ministry of Research and Innovation
NRC: National Research Council, Canada
NSERC: Natural Sciences and Engineering Research Council
OCUR: Ontario Council on University Research
OECD: Organization of Economic Cooperation and Development
SSHRC: Social Sciences and Humanities Research Council
U15: Canadian top 15 research universities
VP: Vice President

## Footnotes

1 The building was partially funded by a $230.3 million loan from Infrastructure Ontario, the arm’s-length government agency that specializes in private-public partnerships (Slaughter 2014).
