# Supplementary material for: 'Innovation policy is a team sport' - insights from non-governmental intermediaries in Canadian innovation ecosystem
Source: Triple Helix (Heidelb). 2018 Nov 27;5(1):8. doi: 10.1186/s40604-018-0062-8 (PMC6404795; doi:10.1186/s40604-018-0062-8)
Supplement: Supplementary file 1 — Translation of the abstract into Arabic.(PDF 41 kb) [file 40604_2018_62_MOESM1_ESM.pdf]

Multilingual abstracts

Arabic

Merli Tamtik

سياسة الابتكار هي نتيجة لعمل جماعي "-لمحات من الوسطاء الغير الحكوميين في النظام الإيكولوجي الكندي للإبداع "

هناك قناعات متزايدة لراسمي السياسات والمهنيين على حد سواء بأنه لا يمكن لنظرية النظام الإيكولوجي للابتكار أن تحقق لوحدها تدفق المعرفة في إطار أنموذج المراوح الثلاثة. يركز هذا التوجه على الطبيعة التعاونية والمترابطة للابتكار، والتي ترتكز على الجوانب الاجتماعية لنقل المعارف التي تدعم العلاقات والشراكات والاتصالات. لم يقع التركيز على الدور الهام للأطراف المتداخلة التي تساعد على تسهيل مثل هذه الشراكات . تبحث هذه الورقة دور ثلاثة أطراف متداخلة في النظام الإيكولوجي الكندي للإبداع وهم المركز الكندي لسياسة البحث العلمي، ومجمع الاكتشاف في آذار/مارس، ونواب رؤساء جامعات البحث. تناولت هذه الدراسة كيفية إنشاء النظم الإيكولوجية، وما هي العوامل التي تؤثر على نجاح الابتكار والجمع بين مختلف الأطراف المتداخلة عبر استجواب 40 خبيراً من الحكومة الاتحادية وحكومات المقاطعات، والمنظمات غير الحكومية، والصناعة، وقطاع التعليم العالي في أونتاريو. تشير النتائج إلى أن الرؤية السياسية والقيادة الناجعة، انتهاج رؤية شاملة لتحديد احتياجات مختلف الأطراف المتداخلة، والوضوح في طرق القياس والابتكار تمثل عوامل هامة في النظام الإيكولوجي الكندي للابتكار.

الكلمات المفتاح: النظام الإيكولوجي للابتكار ، الأطراف المتداخلة، الأطراف الغير حكومية ، كندا
